# Revisiting a historic human brain with magnetic resonance imaging – the first description of a divided central sulcus

**DOI:** 10.3389/fnana.2014.00035

**Published:** 2014-05-19

**Authors:** Renate Schweizer, Gunther Helms, Jens Frahm

**Affiliations:** ^1^Biomedizinische NMR Forschungs GmbH am Max-Planck-Institut für Biophysikalische ChemieGöttingen, Germany; ^2^MR Forschung in der Neurologie und Psychiatrie, Abteilung Kognitive Neurologie, UniversitätsmedizinGöttingen, Germany

**Keywords:** cerebral cortex, cortical anatomy, *pli de passage fronto-parietal moyen*, Conrad Heinrich Fuchs, Carl Friedrich Gauss

## Abstract

In 1860 and 1862, the German physiologist Wagner published two studies, in which he compared the cortical surfaces of brain specimens. This provided the first account of a rare anatomical variation – bridges across the central sulci in both hemispheres connecting the forward and backward facing central convolutions in one of the brains. The serendipitous rediscovery of the preserved historic brain specimen in the collections at Göttingen University, being mistaken as the brain of the mathematician C.F. Gauss, allowed us to further investigate the morphology of the bridges Wagner had described with magnetic resonance imaging (MRI). On the historic lithograph, current photographs and MRI surface reconstructions of the brain, a connection across the central sulcus can only be seen in the left hemisphere. In the right hemisphere, contrary to the description of Wagner, a connecting structure is only present across the post-central sulcus. MRI reveals that the left-hemispheric bridge extends into the depth of the sulcus, forming a transverse connection between the two opposing gyri. This rare anatomical variation, generally not associated with neurological symptoms, would nowadays be categorized as a divided central sulcus. The left-hemispheric connection seen across the post-central sulcus, represents the very common case of a segmented post-central sulcus. MRI further disclosed a connection across the right-hemispheric central sulcus, which terminates just below the surface of the brain and is therefore not depicted on the historical lithography. This explains the apparent inconsistency between the bilateral description of bridges across the central sulci and the unilateral appearance on the brain surface. The results are discussed based on the detailed knowledge of anatomists of the late 19th century, who already recognized the divided central sulcus as an extreme variation of a deep convolution within the central sulcus.

## INTRODUCTION

Wagner (1862), a physiologist at the University of Göttingen, published a book with two “preparatory studies,” in which he described and discussed different measures obtained from *ex vivo* brains of “intelligent men” (Wagner, 1860) and of “microcephalics” in comparison to “normal people” and “quadrumanes”. In his second study Wagner described an especially convoluted brain in which the pre and post-central gyri are connected by bridges across the central sulcus. The present study focuses on this first description of what is now known as a divided central sulcus, a rare and largely unknown anatomical variation.

Wagner’s publications reflect two prevailing aspects of neuroanatomical approaches of his time. The first, introduced by Wagner himself (Bentivoglio, 1998), was the investigation of *ex vivo* brains of distinguished individuals, such as famous scientists, composers, physicians, statesmen, philosophers, and writers (Spitzka, 1907; Hall et al., 1928; Vogt, 1929), with the intention to find measures that would correlate with the exclusive characteristics of the deceased individuals. This approach, although pursued well into present time (Falk et al., 2013), is clearly to be seen within its historic context (Bentivoglio, 1998; Vein and Maat-Schieman, 2008) and remains not without controversy (Hagner, 2003, 2004).

The second aspect concerns the quantitative measures applied to characterize the human brain. The scientific investigation of the human brain during the early 19th century largely consisted of measuring the total weight and volume. The brain was seen as a homogeneous entity notwithstanding the seemingly arbitrary pattern of convolutions on its surface. Realizing the limitations of these measures, anatomists then advanced toward a more detailed depiction of brain surfaces and cerebral convolutions, searching for more meaningful features to quantify individual brains (Clarke and Dewhurst, 1996). The insight that cortical fissures and convolutions indeed form a systematic pattern provided the foundation for a standardized nomenclature of sulci and gyri. A systematic description and naming of major sulci and brain areas was not undertaken until the mid 19th century, with the notable exception of the fissure of Sylvius described already in 1641 (Clarke and Dewhurst, 1996). The fissure of Rolando (the central sulcus) was named in 1839 by the French anatomist Leuret (Broca, 1888). Arnold introduced the concept of the “frontal,” “parietal,” “temporal,” and “occipital” lobes in 1838 which was redefined by Gratiolet in 1857, who established the fissure of Rolando as the posterior limit of the frontal lobe (Broca, 1888).

Within this conceptual framework of precise anatomical descriptions of the convolutions of the brain, Wagner published an example of an especially convoluted brain in his second study (1862). The figure caption identifies it as the brain of Conrad Heinrich Fuchs (1803–1855), a then distinguished and well-regarded physician and pathologist at the University of Göttingen. Wagner (1862) describes the “convolutions at both sides of the central sulcus of that brain as being unequal, the forward facing being more marked on both hemispheres than the backward facing, both with many deep folds and twists, so that they appear as discontinuous.” He also states that “both [central sulci] are connected by bridges, the one on the left side being very prominent, rising with a wide root out of the forward-facing central convolution” (translation by the authors). Wagner did not comment further on this connection between the pre- and the post-central gyrus. It was only later that anatomists referred to Wagner as being the first to describe this rare anatomical variation of the central sulcus (Heschl, 1877; Ebenstaller, 1884; Broca, 1888; Cunningham, 1890; Retzius, 1896; Spitzka, 1902, 1907; Waterston, 1907; Symington and Crymble, 1913). To our knowledge, there is only one published report within the last 60 years, presenting structural and functional MRI data of an individual who exhibits a divided central sulcus in the left hemisphere (Alkadhi and Kollias, 2004). This report brought to our attention the lithograph of the brain in Wagner (1862) as the first historical observation of this rare variation.

The serendipitous discovery of this specific brain specimen in the collections of the University of Göttingen was based on the fact that it has been mistaken as the brain of the mathematician C. F. Gauss (1777–1855) – probably for a long time (Schweizer et al., 2014). Both brains were part of Wagner’s (1860, 1862) studies and kept in the University’s collections since then. The existence of a prominent and distinct bridge across the left central sulcus on the documentary magnetic resonance imaging (MRI) of the brain of C. F. Gauss (Wittmann et al., 1999; Frewer and Hanefeld, 2000) recently raised suspicion about the true identity of the brain. Subsequent comparisons with the detailed lithographs of the explicitly labeled brain surfaces in Wagner’s (1860, 1862) publications ultimately allowed for a correct assignment of the identities of the brains of C. H. Fuchs and C. F. Gauss.

State-of-the-art three-dimensional MRI of the historic brain specimen of C. H. Fuchs was then performed, allowing a complete assessment of connecting structures across the central sulci beyond their appearance on the brain surface. This was pursued to answer the question if the bridges described by Wagner have the same anatomical structure as the divided central sulci reported by later anatomists. Closer inspection of the brain surface of C. H. Fuchs in Wagner (1862) also raised questions about the exact location of the right-hemispheric bridge, which appeared to run across the post-central rather than the central sulcus, contrary to Wagner’s description.

## MATERIALS AND METHODS

The brain of C. H. Fuchs was separated into three parts, the two hemispheres and the cerebellum with brainstem, by incisions across the midbrain and the corpus callosum. It was kept in a lidded glass jar in the University collections, now in the Institute for Ethics and History of Medicine at the University Medical Center Göttingen. In the jar, the hemispheres and the cerebellum were placed on gauze. The details of the original fixation process are unknown. In his thesis, Wagner (1864), a son of Rudolph Wagner, stated that the brain specimens were fixed in alcohol. This liquid was replaced by 4% formalin in 1998 (Wittmann et al., 1999) and again in 2011 (Wittmann, personal information). Photographs of the brain were taken at the MediaService of the Max-Planck-Institute for biophysical Chemistry, Göttingen. The separated hemispheres and cerebellum were taken out of the fixative, arranged in a natural position and photographed in a view closely matching the lithograph in the publication of Wagner (1862).

MRI was performed at the Department of Cognitive Neurology, MR Research in Neurology and Psychiatry, University Medical Center Göttingen, on a 3T clinical whole-body MRI System (Tim Trio Siemens Healthcare, Erlangen, Germany) using an 8-channel head coil with approval by the ethics committee of the University Medical Center. For the MRI examination, the brain was assembled in its natural position and submerged in distilled water to obtain better contrast at the brain surface. Eight 3D images were acquired using a 3D multi-echo FLASH sequence (Frahm et al., 1986; TR = 23 ms, eight echoes at equidistant echo times TE = 2.46, 4.96, ..., 19.68 ms with a bandwidth of 500 Hz/pixel, flip angle = 20°, resolution = 0.5 mm × 0.5 mm × 0.5 mm, 7/8 partial Fourier acquisition, total measurement time = 22 min). Because of the short T1 of the specimen (150–300 ms), the chosen combination of TR and flip angle yielded a proton-density weighted contrast, so that the cortical surface presents with a marked contrast against the largely suppressed signal of the surrounding water. Images were averaged to increase the signal-to-noise-ratio as well as to reduce distortions at the air-water interfaces (Helms and Dechent, 2009). The proton density contrast was augmented by a mild T2^*^ weighting at an effective TE of 11 ms (Helms et al., 2011). The cortical surface was rendered at a threshold of 50 to a depth of 9 pixels and rotated to match the photographic perspective using the freeware MRIcro viewer (www.cabiatl.com/mricro/mricro/mricro.html).

## RESULTS

**FIGURE 1:**
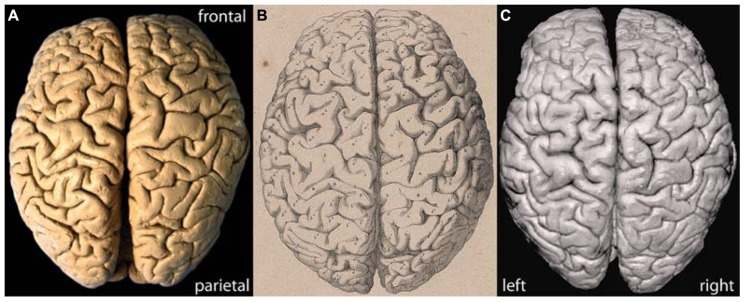
**(A)** Actual photograph, **(B)** historic lithograph (Wagner, 1862), and **(C)** MRI surface reconstruction of the brain of C. H. Fuchs.

**Figure 1** shows a photograph of the brain of C. H. Fuchs in comparison with the lithograph published by Wagner (1860; **Figures 1A,B**). The surface pattern of the convolutions in the lithograph and photograph fully match and the individual sulci and gyri can clearly be identified on both images. The slight deviation in the alignment of the separated hemispheres in the photographs is due to a slight deformation of the left hemisphere along the midsagittal surface, probably a consequence of the long storage. The surface reconstruction based on 3D MRI (**Figure 1C**) allows for a detailed comparison with the lithograph and photograph and confirms the adequacy and quality of the applied MRI methods.

**FIGURE 2:**
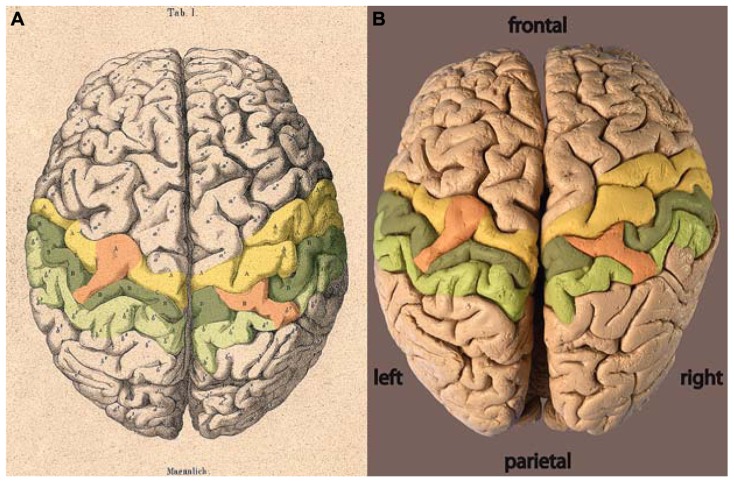
**(A)** Lithograph and **(B)** photograph of the brain of C. H. Fuchs (Wagner, 1862) with areas highlighted: precentral gyrus = yellow, post-central gyrus = dark green, parietal gyrus = light green, bridges = orange.

The locations of the two bridges described by Wagner (1860) are highlighted by color on each hemisphere in **Figure 2**. From Wagner’s original labeling it can be inferred that the bridge on the left hemisphere joins the precentral gyrus across the central sulcus to the post-central gyrus. On the right hemisphere, the bridge reaches out of the post-central gyrus across the post-central sulcus toward the parietal gyrus. No comparable bridge is seen on the brain surface across the right-hemispheric central sulcus.

**FIGURE 3:**
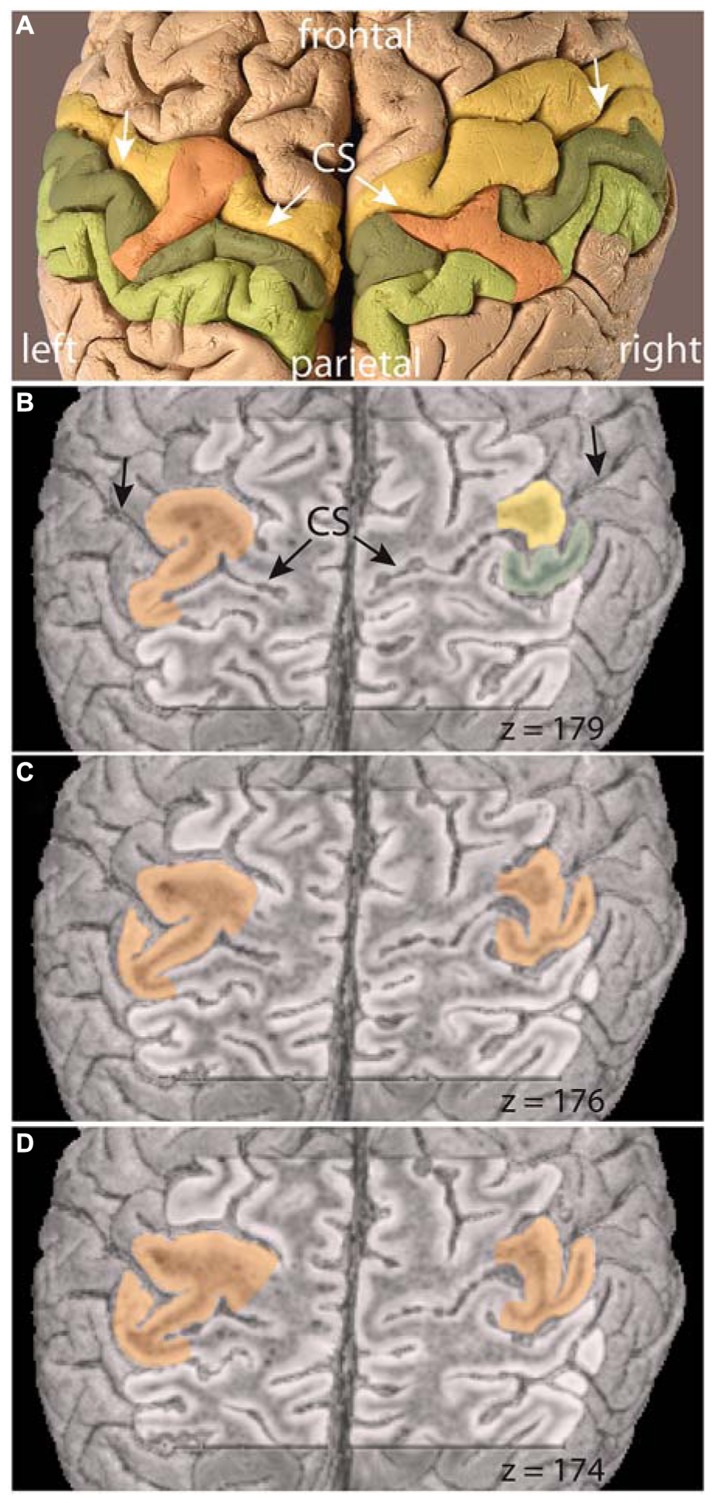
**(A)** Photograph and **(B–D)** cross-sectional MRI views of the brain of C. H. Fuchs at mid-depth level of the central sulci (CS, arrows). *z* = z-coordinate in mm, precentral gyrus = yellow, post-central gyrus = dark green, bridges = orange.

The 3D MRI dataset not only offers a surface reconstruction as shown in **Figure 1C**, but also allows for anatomic views at different depths below the brain surface. **Figure 3** provides cross-sections at different levels, focusing on the central sulcus. The upper level was chosen to be in the area of the left-hemispheric bridge just below the brain surface (**Figure 3B**: *z* = 179). In the left hemisphere, a prominent connection between the pre- and post-central gyrus can be seen, congruent with the surface view of the bridge. In the right hemisphere, however, the pre- and post-central gyrus are very close, but nevertheless clearly separated, as expected from the usual course of the central sulcus.

The following two sections (**Figure 3C**: *z* = 176; D: *z* = 174) present deeper levels, the lowest at approximately mid-depth of the central sulcus. In the left hemisphere, the prominent connection between the pre- and post-central gyri persists on both deeper levels. Thus, Wagner’s term bridge is adequate only in the sense that there is a connection of the two distinct gyri across a sulcus. However, the connection is not separated below the surface (as the term bridge would imply), but persists continuously deeply into the central sulcus. Thereby, the central sulcus is divided in an upper part pointing toward the interhemispheric fissure and a lower part pointing toward the Sylvian fissure.

**FIGURE 4:**
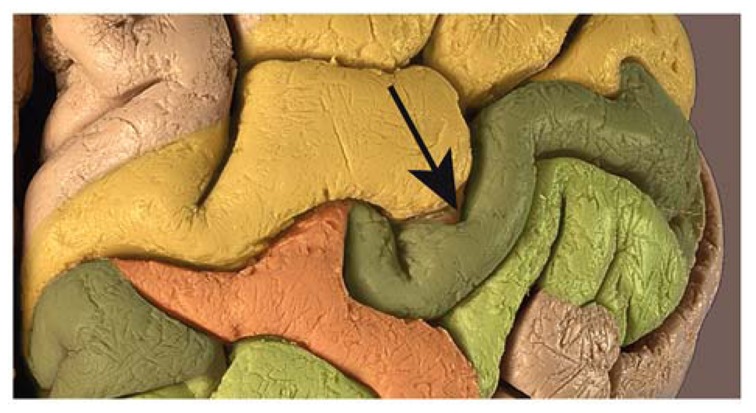
**Photograph of the right central sulcus of the brain of C. H. Fuchs, showing the respective elevated *pli de passage* within the sulcus (arrow) which does not reach the surface of the brain.** Precentral gyrus = yellow, post-central gyrus = dark green, bridges = orange.

The two deeper level views reveal a previously undescribed connection of the pre- and post-central gyrus in the right hemisphere. The connection between these two gyri across the central sulcus extends from the fundus to a very high level, but does not reach the surface. At the deepest level (**Figure 3D**: *z* = 174) its appearance is equivalent to the connection in the left hemisphere, dividing the central sulcus in two parts. At the midlevel cross-section (**Figure 3C**: *z* = 176), the connection presents itself as a band of gray-matter, but no white matter can be seen, analogous to the crown of a connecting gyrus. The upper level (**Figure 3B**: *z* = 179) then shows the separate pre- and post-central gyri and no bridge across the central sulcus (**Figure 2**). An enlargement of the photograph at the appropriate location along the central sulcus reveals that the crown of the connection can be seen just below the brain surface (**Figure 4**).

**FIGURE 5:**
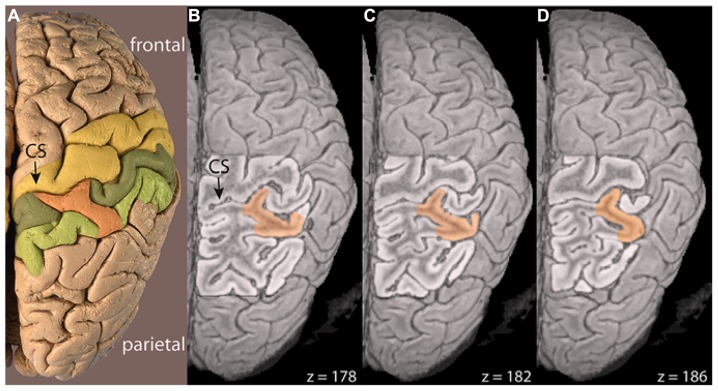
**(A)** Photograph and **(B–D)** cross-sectional MRI views of the right-hemispheric brain of C. H. Fuchs cutting into the depth of the central sulcus (CS, arrow). *z* = z-coordinate in mm, post-central gyrus = dark green, parietal gyrus = light green, bridges = orange.

The prominent bridge on the surface of the right hemisphere clearly stems from the post-central gyrus and crosses the post-central sulcus. The sections covering the right central and post-central sulcus (**Figure 5**) illustrate that the connection persists in all three slices (*z* = 178, 182, 186) confirming that the bridge is not only on the brain surface, but extends into the depth of the post-central sulcus.

## DISCUSSION

The present MRI study revisits the historic brain specimen of C. H. Fuchs, which was the basis for the first description of the rare anatomical variation of a bridged or divided central sulcus. 3D MRI demonstrates that the connection across the pre- and post-central gyrus is not only present on the surface, but forms a continuous structure from the surface deeply into the fundus. This variation results in a completely divided central sulcus with a shorter upper part running toward the interhemispheric fissure and a longer lower part toward the Sylvian fissure. MRI additionally revealed a connection between the pre- and post-central gyrus in the right central sulcus. This connection rises from the depth of the central sulcus, but does not reach the surface and is therefore not visible on the brain’s surface. In addition the MRI data confirm the connection across the post-central sulcus on the surface of the right hemisphere.

The significance of Wagner’s work is the detailed description of the convolutions on the cortical surface and, in the historic context, their unusually precise illustration by his lithographers H. Loedel and G. Honig. The description of the bridges across the central sulcus connecting the pre- and post-central gyri was a result of his detailed investigations. But since Wagner’s focus was on the convolutions of the brain surface he did not study the underlying morphology of this anatomical variation. Fortunately this “superficial” interest led to the preservation of the complete specimen, which – contrary to other famous brains, like Einstein’s or Lenin’s – was not cut into pieces.

After Wagner, Heschl (1877), Broca (1888), and Cunningham (1890) were the first to provide a more detailed insight into the specific anatomy and the possible origin of the bridged central sulcus. Heschl (1877) adressed, in a short but insightful publication, the importance of “deep convolutions,” analog to the *pli de passage* [Gratiolet (1854), cited in Regis et al., 2005], being hidden in the depth of all major sulci, but nevertheless determining the individual course of the sulci on the brain surface. This concept was recently used by Regis et al. (2005) in a sulcal root model, stressing the importance of deep convolutions on the individual brain folding patterns. Heschl then specifically focuses on the deep convolution between the upper and middle third of the central sulcus, where the precentral gyrus exhibits a convex posterior bend. He states that this deep convolution can vary in its extent toward the brain surface and mentions that the “bridged” central sulcus in the brain of C. H. Fuchs (Wagner, 1862) can be explained by a deep convolution of the central sulcus extending up to the brain surface (Heschl, 1877).

Broca (1888) also discusses this variation of the central sulcus in his comprehensive “Memoires d’Anthropologie.” Within the central sulcus, running from the interhemispheric fissure down to the Sylvian fissure, he defines two bends, the superior genu and inferior genu, both anteriorly convex with a concavely oriented section in between. Broca also describes a connection between the pre-central and the post-central gyri at or below the superior genu which he calls the *pli de passage fronto-parietal moyen* (middle). This connection is hidden in the depth of the central sulcus and equivalent to the deep convolution described by Heschl (1877). Broca (1888) reports that this connection always runs across the fundus of the central sulcus, he has never seen it rising to the surface, except in one brain; the brain of an “idiot,” which was generally disturbed by a great number of severe abnormalities. In retrospect, Wagner’s term “bridge” proved incompatible with the underlying morphology. Our MRI data clearly corroborate the terminology chosen by Broca (*pli* meaning crease or fold).

The development of a divided central sulcus can be related to the individual fetal ontogeny. According to Cunningham (1890), the central sulcus develops out of two distinct parts being separated by an elevation. The upper part later becomes the upper third and the lower part the lower two-thirds of the fully formed central sulcus. Later in development, a faint furrow appears on that elevation, partially uniting the two originally separated portions. The two parts then merge and a deep annectant gyrus remains in the fundus of the central sulcus as a residue of the separating elevation. In the rare case of a divided central sulcus, the two original portions remain distinct and the intervening elevation remains on the surface. A divided central sulcus can therefore be seen as an incompletely developed central sulcus, in which the elevation between the two original portions, usually buried in the depth of the sulcus, stays on the surface of the brain (Cunningham, 1890).

Up to the present day, only singular cases of a divided central sulcus have been reported, often with the additional citation of other single case reports [Spitzka (1902): one reported, 13 cited; Waterston (1907): one reported, three cited including Spitzka (1902), Alkadhi and Kollias (2004): one report]. Data on the general incidence of a divided central sulcus are sparse as only four studies report the number of incidences in relation to the number of investigated brains. Heschl (1877) presented the largest data base on this topic. As a prosector of the general hospital in Vienna, he examined 1087 brains within less than 1 year. He reports five unilaterally bridged central sulci in 632 male brains and one unilaterally bridged central sulcus in 455 female brains, yielding an approximate ratio of 1:130 for males and 1:455 for females. Ebenstaller (1884) reported two cases in about 200 observed brains, Symington and Crymble (1913) reported one case in 237 hemispheres (116 brains) and Retzius (1896) reported no case within the 100 adult brains he studied. Based on Retzius (1896) and Ebenstaller (1884), Ono et al. (1990) assumes that an unilateral interrupted central sulcus can be found in 1% of the cases, while the total number of studies cited above would point to an incidence rate of about 0.6%.

Wagner (1862) described bridges in both hemispheres, however, the lithograph depicting the brain surface does not show a connection across the right central sulcus, but rather across the post-central sulcus. Our MRI data show that this connection extends from the brain surface to the sulcal fundus, it can therefore be identified as an interruption of the post-central sulcus. Such a variation is very common as 40% of the right hemispheres show an interrupted (two segments) post-central sulcus (Ono et al., 1990) in striking contrast to the incidence (<1%) for an interrupted central sulcus.

The present MR images further reveal that a transverse connection between the precentral and the post-central gyrus is also present across the right-hemispheric central sulcus. Terminating just below the brain surface, this *pli de passage fronto-parietal moyen* is very high but less complete than the one on the left hemisphere. Thus, if exclusively relying on the historic lithograph, the described “bridges” across the central sulci can only be verified on the left hemisphere. Accordingly, in the literature citing Wagner’s finding, (Heschl, 1877; Ebenstaller, 1884; Broca, 1888; Cunningham, 1890; Retzius, 1896; Waterston, 1907) none of the authors mention the bilaterality of the bridges and Spitzka (1902) even explicitly points out a unilateral bridge.

Due to the availability of the well-preserved historic specimen for state-of-the-art MRI we uncovered the existence of a right-hemispheric connection across the central sulcus below the brain surface. This explains the apparent inconsistency between Wagner’s description of bilateral bridges across the central sulci and the lithograph and MRI-based surface reconstruction showing only a bridge in the left hemisphere. Although the right-hemispheric connection does not quite reach the brain surface, it may well have been recognized by an anatomist handling the specimen. In fact, a guided inspection of the photograph in **Figure 4** reveals its presence, thus adding support to the hypothesis that Wagner was indeed describing this bridge.

In summary, we conclude that the preserved brain of C. H. Fuchs exhibits the rare variation of a *pli de passage fronto-parietal moyen* complete in the left hemisphere, which results in a divided central sulcus clearly visible on the brain surface. MRI of the historic brain specimen revealed that the *pli de passage fronto-parietal moyen* is incomplete in the sense that the transverse connection does not extend to the brain surface and can therefore not be seen in the lithograph or MRI-based surface reconstruction. The connection seen on the brain surface of the right hemisphere is across the post-central sulcus and represents a segmented post-central sulcus.

## Conflict of Interest Statement

The authors declare that the research was conducted in the absence of any commercial or financial relationships that could be construed as a potential conflict of interest.
